# Increased Risk of Paroxysmal Atrial Fibrillation Episodes Associated with Acute Increases in Ambient Air Pollution

**DOI:** 10.1289/ehp.8371

**Published:** 2005-09-20

**Authors:** David Q. Rich, Murray A. Mittleman, Mark S. Link, Joel Schwartz, Heike Luttmann-Gibson, Paul J. Catalano, Frank E. Speizer, Diane R. Gold, Douglas W. Dockery

**Affiliations:** 1 Department of Environmental Health, Harvard School of Public Health, Boston, Massachusetts, USA; 2 Department of Epidemiology, Harvard School of Public Health, Boston, Massachusetts, USA; 3 Beth Israel Deaconess Medical Center and Harvard Medical School, Boston, Massachusetts, USA; 4 New England Medical Center, Tufts University, Boston, Massachusetts, USA; 5 Channing Laboratory, Brigham and Women’s Hospital and Harvard Medical School, Boston, Massachusetts, USA; 6 Department of Biostatistics, Harvard School of Public Health, Boston, Massachusetts, USA; 7 Department of Biostatistical Science, Dana-Farber Cancer Institute, Boston, Massachusetts, USA

**Keywords:** air pollution, arrhythmias, fibrillation, epidemiology, case—crossover, ozone

## Abstract

Objectives: We reported previously that 24-hr moving average ambient air pollution concentrations were positively associated with ventricular arrhythmias detected by implantable cardioverter defibrillators (ICDs). ICDs also detect paroxysmal atrial fibrillation episodes (PAF) that result in rapid ventricular rates. In this same cohort of ICD patients, we assessed the association between ambient air pollution and episodes of PAF.

Design: We performed a case–crossover study.

Participants: Patients who lived in the Boston, Massachusetts, metropolitan area and who had ICDs implanted between June 1995 and December 1999 (*n* = 203) were followed until July 2002.

Evaluations/Measurements: We used conditional logistic regression to explore the association between community air pollution and 91 electrophysiologist-confirmed episodes of PAF among 29 subjects.

Results: We found a statistically significant positive association between episodes of PAF and increased ozone concentration (22 ppb) in the hour before the arrhythmia (odds ratio = 2.08; 95% confidence interval = 1.22, 3.54; *p* = 0.001). The risk estimate for a longer (24-hr) moving average was smaller, thus suggesting an immediate effect. Positive but not statistically significant risks were associated with fine particles, nitrogen dioxide, and black carbon.

Conclusions: Increased ambient O_3_ pollution was associated with increased risk of episodes of rapid ventricular response due to PAF, thereby suggesting that community air pollution may be a precipitant of these events.

In previous studies, we reported statistically significant associations between ambient air pollution and cardiac arrhythmias in patients with implantable cardioverter defibrillator (ICD) devices (Dockery et al. 2005a, 2005b; Peters et al. 2000b; Rich et al. 2005). A pilot study of 100 patients in Boston, Massachusetts, found significantly increased risk of ICD discharges associated with nitrogen dioxide and black carbon among patients with repeated events (Peters et al. 2000b). In a larger study of approximately 200 Boston-area ICD patients, we found a nonstatistically significant increased risk of ventricular arrhythmias (confirmed by an electrophysiologist) associated with 2-day mean NO_2_, particulate matter < 2.5 μm in aerodynamic diameter (PM_2.5_), black carbon, carbon monoxide, ozone, and sulfur dioxide (Dockery et al. 2005a, 2005b). In a case–crossover analysis of these data, which allowed us to match the time of onset of these arrhythmias with ambient air pollution concentrations, we found stronger, statistically significant associations of ventricular arrhythmias with mean PM_2.5_ and O_3_ in the 24 hr before the arrhythmia (Rich et al. 2005).

Although ICDs are designed to detect and treat life-threatening ventricular arrhythmias, supraventricular arrhythmias may also be detected. Many of these supraventricular arrhythmias may be atrial fibrillation, which is the most common sustained arrhythmia in clinical practice (Go et al. 2001) and a risk factor for stroke (Prystowsky et al. 1996) and premature mortality (Kanel et al. 1983). We used a case–crossover design to examine the association of ICD-detected paroxysmal atrial fibrillation and hourly measurements of community air pollution concentrations.

## Materials and Methods

### Study population.

Two hundred three patients who had a third-generation Guidant ICD (Cardiac Pacemakers, Inc., Minneapolis, MN) implanted at the Tufts–New England Medical Center between 1 June 1995 and 31 December 1999, were followed until their last clinic visit before 15 July 2002. Patients who lived within 40 km (25 mi) of the air pollution monitoring station at the Harvard School of Public Health were included for analysis. The Guidant ICDs record intracardiac electrograms and were the most common ICD implanted at Tufts–New England Medical Center during the study period. Each patient’s first 14 days after implantation and any events that occurred during inpatient hospital visits were excluded. Further description of this population has been published previously (Dockery et al. 2005a, 2005b).

### Outcome and clinical data.

For each ICD-recorded episode of tachyarrhythmia, the date, time, beat-to-beat intervals, and intracardiac electrogram before, during, and after episodes were recovered from the ICD. In a small number of cases in which the patient experienced a large number of ICD-detected episodes since the previous clinic visit, early electrograms in the ICD memory, but none of the other episode-specific data, may have been overwritten. ICD settings including ventricular tachycardia rate cutoffs (i.e., detection rates) were also abstracted from the ICD records. Ventricular tachycardia rate cutoffs were set by the treating electrophysiologist based on the clinical features of the patients.

All of the ICD-detected episodes were reviewed and characterized by an electrophysiologist (M.S.L) blinded to air pollution levels. Details of this arrhythmia classification have been published previously (Dockery et al. 2005b). Briefly, patients who presented with atrial fibrillation at all clinic follow-ups were classified as in permanent atrial fibrillation, and they were excluded from this analysis. Episodes of paroxysmal atrial fibrillation (PAF) were defined by a ventricular rate between 120 and 200 beats per minute, irregularity of the beat-to-beat intervals, no change in QRS morphology (except for a small number of cases with no ventricular electrogram), and lack of conversion following ventricular therapies (except when therapy was not applied). If a dual-chamber device had been implanted and an atrial electrogram was available, the atrial electrogram was also used to characterize ICD-recorded episodes. This analysis was restricted to PAF episodes that occurred at least 60 min after the previous event. Residence ZIP code, date of birth, race/ethnicity, clinic visit dates, and medications prescribed (beta-blockers, digoxin, and other antiarrhythmics) were abstracted from patients’ records.

The Harvard School of Public Health Human Subjects Committee and the Tufts–New England Medical Center Institutional Review Board approved this record review study.

### Air pollution.

The air pollution measurements have been described previously (Dockery et al. 2005a, 2005b; Rich et al. 2005). Briefly, ambient concentrations of O_3_, NO_2_, SO_2_, and CO were measured hourly by the Massachusetts Department of Environmental Protection at four to six sites in the Boston metropolitan area during the entire follow-up period. We calculated the hourly average air pollution concentration across all available monitoring stations, accounting for differences in the annual mean and daily standardized deviations of each monitor (Schwartz 2000). PM_2.5_ was measured hourly in South Boston (~ 5 km east of the Harvard School of Public Health) from 1 April 1995 to 20 January 1998, and at the Harvard School of Public Health from 16 March 1999 to 31 July 2002. Black carbon was measured hourly in South Boston from 1 April 1995 to 29 March 1997, and at the Harvard School of Public Health from 15 October 1999 to 31 July 2002.

### Acute effect of pollutants.

We analyzed the association of ambient air pollution concentrations and episodes of PAF using a case–crossover design (Maclure 1991). These methods have been used previously to study triggers of acute cardiovascular events (Albert et al. 2000; D’Ippoliti et al. 2003; Hallqvist et al. 2000; Mittleman et al. 1995; Peters et al. 2000a; Rich et al. 2005). In this design, each subject contributes information as a case during the event periods and as a matched control during nonevent times. Because cases and their matched controls are derived from the same person and a conditional analysis is conducted, non-time-varying potential confounders such as underlying medical condition and long-term smoking history are controlled by design. Variables that may be related to both air pollution and the occurrence of PAF that fluctuate over time (e.g., meteorologic conditions) are possible confounders.

We defined case periods by the detection time of each confirmed episode of PAF, rounded to the nearest hour. We matched control periods on weekday and hour of the day within the same calendar month (Lumley and Levy 2000). We calculated average pollution concentrations and weather conditions during the hour and during the 24 hours before the case and control time periods for this analysis.

Conditional logistic regression models, including the mean pollutant concentration in the hour of the arrhythmia (lag hour 0) and natural splines [3 degrees of freedom (df)] for the mean temperature, dew point, and barometric pressure in the 24 hr before the arrhythmia, were run separately for each pollutant (PM_2.5_, black carbon, NO_2_, CO, SO_2_ and O_3_). Different individuals may have different cardiac responses to pollution, based on their clinical history and genetic characteristics. Therefore, we included a frailty term (Therneau and Grambsch 2000) for each subject (akin to a random intercept) in all the above models. Odds ratios (ORs), 95% confidence intervals (CIs), and *p*-values for statistical significance testing are presented for an interquartile range increase in each pollutant. We considered associations with longer exposures before the PAF episode using the mean of the pollutant in the previous 24 hr (lag hours 0–23).

To assess the sensitivity of our results to the influence of outliers, we reran analyses, trimming the highest 5% and lowest 5% of air pollution concentrations. For O_3_, which has a strong seasonal pattern, we examined whether the association between PAF and O_3_ concentration was limited to the 6 months with the highest mean ambient temperature (May–October) by adding an O_3_/warm month interaction term to the conditional logistic regression model. We assessed the linearity of the PAF and O_3_ association by replacing the linear air pollution term with a penalized spline (3 df) in the conditional logistic regression model. We plotted the covariate adjusted log OR for the risk of PAF in the spline and linear models versus 1-hr O_3_ concentration.

We used SAS (version 9.1; SAS Institute Inc., Cary, NC) software to construct all datasets and to calculate descriptive statistics. We used S-Plus 6.2 (Insightful Inc., Seattle, WA) software for all modeling.

## Results

There were 203 ICD patients enrolled in the study who lived within 40 km of the Harvard School of Public Health with a mean (± SD) follow-up time of 3.1 ± 1.8 years (maximum = 7.0 years). Ninety-five patients had a total of 1,574 recorded ICD events, 933 of which were separated by > 1 hr. Ninety-one (9.8 %) of these events, among 29 subjects, were confirmed episodes of PAF. Because PM_2.5_ and black carbon were not measured during the entire study period, analyses of PM_2.5_ included at most 52 episodes of PAF from 22 subjects, and analyses of black carbon included at most 46 episodes of PAF from 18 subjects.

The 29 subjects with PAF episodes were primarily male (79%) and white (79%), and they ranged in age from 45 to 78 years (mean, 65 years). At their first clinic follow-up visit, 69% of subjects were listed as being prescribed beta-blockers, 57% digoxin, and 24% other antiarrhythmics (i.e., amiodarone, quinidine, sotalol, or mexilitine). Two subjects (7%) were not prescribed any of these medications. The most common diagnoses at implantation were coronary artery disease (76%) and idiopathic cardiomyopathy (22%). Before ICD implantation, 55% of subjects had left ventricular ejection fractions < 35%. Subjects’ ICDs were programmed with ventricular tachycardia detection rates (i.e., ventricular rate threshold above which the electrogram and date/time for a tachyarrhythmia would be recorded) that had a 10th to 90th percentile range of 140 to 200 beats/min (median = 175).

Of the 29 subjects who experienced at least one episode of PAF, 15 (52%) experienced > 1 event, while 2 (7%) experienced ≥ 10. Twenty (69%) also experienced a ventricular arrhythmia during follow-up. Episodes of PAF were more frequent in the late morning (0900–1100 hr), with a smaller evening peak (1800–2000 hr).

The distributions of air pollution concentrations and meteorologic characteristics in Boston during the study period, averaged hourly and daily, are summarized in Table 1. The highest average PM_2.5_ and black carbon concentrations were observed early in the morning (0600–0800 hr), highest NO_2_ in the early morning (0600–0800 hr) and early evening (1600–2100 hr), and highest O_3_ at midday (1200–1400 hr). Further detail has been provided previously (Dockery et al. 2005b).

We found a statistically significant increased risk of PAF associated with mean O_3_ concentration in the concurrent hour (lag hour 0; Table 2). The estimated relative odds for the 24-hr moving average concentration was positive (OR > 1), but not statistically significant. We did not find statistically significant associations with any other pollutant in the concurrent hour, but associations were positive for PM_2.5_ and NO_2_. Risk estimates for 24-hr average PM_2.5_, NO_2_, and black carbon were positive, but none was statistically significant. Risk estimates for 24-hr average CO and SO_2_ were protective (OR < 1), but neither was statistically significant (Table 2).

For O_3_ in the concurrent hour, there was little change in risk of PAF when we excluded the top 5% and bottom 5% of concentrations (OR = 2.15, 95% CI = 1.04–4.44, *p* = 0.04). The association between PAF and O_3_ in the concurrent hour in the cold months (OR = 2.21; 95% CI = 0.98–4.98; *p* = 0.06) was comparable to that in the warm months (OR = 1.98; 95% CI = 1.05–3.73; *p* = 0.04), with no significant interaction (*p* = 0.84).

**Figure 1:**
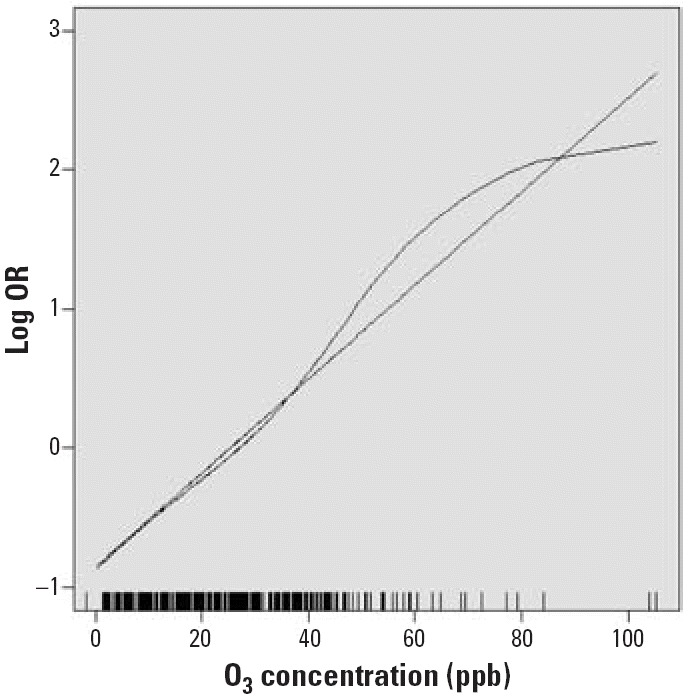
Log OR of PAF by 1-hr O_3_ concentration modeled as a linear term and using a penalized spline with 3 df. Vertical lines on abscissa indicate the O_3_ concentrations of observed events.

Figure 1 shows the covariate adjusted log OR for the risk of PAF versus 1-hr O_3_ concentration modeled using first a linear term and then a penalized spline (3 df). We found no evidence of a deviation from linearity (nonlinear term, *p* = 0.63).

## Discussion

In a study designed to assess the association of ambient air pollution with ventricular arrhythmias among ICD patients, 91 of the ICD-detected episodes were identified by electrophysiologist review as PAF. Although these episodes of PAF were likely an underrepresentation of all those PAF episodes experienced by these patients, they provided a unique opportunity to assess associations between air pollution and episodes of PAF. We found a statistically significant 2-fold increase in risk of PAF episodes associated with each 22-ppb increase in mean ambient O_3_ concentration in the concurrent hour. We found no evidence that this association was nonlinear.

An earlier study reported a 10.5% increase in supraventricular ectopy (~ 3.5 beats/hr increase in supraventricular ectopy compared to the population mean rate of supraventricular ectopy) associated with each 7-μg/m^3^ increase in ambient PM_10_ (particulate matter < 10 μm in aerodynamic diameter) concentration in a panel of chronic obstructive pulmonary disease patients (Brauer et al. 2001). They reported smaller increases in supraventricular ectopy associated with outdoor and personal PM_2.5_ and sulfates.

Our findings identify ambient air pollution as a potential precipitant of supraventricular arrhythmias. Atrial fibrillation is the most common supraventricular arrhythmia. At least 2.3 million adults in the United States have some form of atrial fibrillation (Go et al. 2001), and this number is likely an underestimate because many people with this condition are asymptomatic (Chugh et al. 2001). The incidence of atrial fibrillation doubles with each decade of adult life (Falk 2001). Although atrial fibrillation is not usually considered a lethal rhythm, it is associated with premature mortality and increased risk for hospitalization and stroke (Wolf et al. 1998; Benjamin et al. 1998). If not on antithrombotic therapy, people with atrial fibrillation have a 5-fold increased risk of stroke (Ryder and Benjamin 1999). Therefore, even a modest risk of atrial fibrillation associated with acute exposure to elevated ambient air pollution in the general population would have a substantial attributable risk.

In prior analyses in this cohort of ICD patients, we found significantly increased risk of ventricular arrhythmias associated with mean PM_2.5_ and O_3_ concentrations in the 24 hr before the episode (Rich et al. 2005), and marginally significant increased risk (*p* < 0.10) associated with mean black carbon and NO_2_ concentrations over the previous 2 days (Dockery et al. 2005a, 2005b). The findings of positive associations between episodes of PAF and O_3_ concentration (1-hr) is consistent with these observations, although the timing (1 hr vs. 24 hr or 1 day) suggests a more rapid response to air pollution with PAF.

O_3_ is an acute lung irritant that has been associated with acute myocardial infarction (Ruidavets et al. 2005), decreased lung function, exacerbation of asthma or other respiratory conditions, increased hospitalizations, and premature mortality (Thurston and Ito 1999). O_3_ is a highly reactive oxidant formed by photochemical reactions in the atmosphere. O_3_ concentrations are highest on warm sunny days, and highest during the afternoon hours. However, we found a statistically significant association with O_3_ after adjustment for temperature, and we found no evidence that the O_3_ associations were restricted to the six warmest months.

We also found positive associations with PM_2.5_, NO_2_, and black carbon, but the CIs were wide and the risk estimates were not statistically significant. The number of PAF episodes with matching O_3_ and NO_2_ concentrations was small (*n* = 90), and they were even smaller for PM_2.5_ (*n* = 52) and black carbon (*n* = 46), which resulted in reduced power to detect any associations. Thus, this small number of confirmed PAF episodes dictates caution in our interpretation of specific associations. Although we have highlighted the association with O_3_ in the concurrent hour, it would be premature to attribute the increased risk of PAF to O_3_ alone. We suggest that community air pollution may be associated with the incidence of PAF. Confirmation of this association and examination of associations with specific pollutants requires a larger number of confirmed PAF episodes.

A problem in studying incidence of PAF is the definition of time of onset of new episodes. Although the ICD device provides a detection time for each episode of PAF, this is the time that the ventricular rate (responding to the atrial stimulus) exceeded the patient’s specific programmed criteria for a tachyarrhythmia. The PAF episode may have started earlier than the time recorded by the ICD. This situation would lead to mismatching of air pollution concentrations to case and control time periods. However, this exposure misclassification would be nondifferential with respect to case/control status. Therefore, it would have resulted in a bias toward the null, underestimates of risk, and wide CIs.

Episodes of PAF also may have been misclassified. However, any outcome misclassification, if present, was likely independent of air pollution levels and nondifferential. This misclassification would have produced wider CIs, a bias toward the null, and underestimates of risk.

Our analysis was limited to a subset of all PAF episodes that these subjects experienced. PAF episodes with ventricular response rates that remained below the ICD’s preset detection criteria for the duration of the arrhythmia would not have been recorded. These under-detected episodes likely represented a substantial fraction of the PAF episodes experienced by these patients. However, we used the case–crossover method, where each person serves as his or her own control, and event times are contrasted with matched control times. Such misclassification would have resulted in a loss of power, but no bias in our risk estimates. Whether our finding of an association between transient ambient air pollution concentrations and PAF is limited to this particular subset of PAF episodes, however, is unknown. New studies using devices programmed to detect a wider range of PAF episodes with more precise data on the timing of arrhythmia initiation are required to confirm and quantify this association further.

## Figures and Tables

**Table 1:** Boston air pollution profile, August 1995 to June 2002.

		Percentile			
Parameter	No. of hours or days	25th	50th	75th	Maximum
PM_2.5_ (μg/m^3^)^a^					
Hourly	48,592	5.6	9.2	15.0	84.1
Daily	2079	6.7	9.8	14.5	53.2
Black carbon (μg/m^3^)^b^					
Hourly	36,789	0.44	0.77	1.35	23.93
Daily	1555	0.58	0.94	1.41	7.32
NO_2_ (ppb)					
Hourly	60,555	15.8	21.7	29.0	78.8
Daily	2526	18.1	22.4	27.3	61.8
SO_2_ (ppb)					
Hourly	60,620	2.6	4.3	7.5	71.6
Daily	2526	3.2	4.8	7.3	31.4
CO (ppm)					
Hourly	60,091	0.46	0.73	1.04	5.83
Daily	2526	0.52	0.78	1.03	2.48
O_3_ (ppb)					
Hourly	60,210	11.3	22.2	33.0	119.5
Daily	2524	15.2	22.6	30.9	77.5
Temperature (°C)					
Hourly	60,449	3	11	18	36
Daily	2526	4	11	18	32
Dew point (°C)					
Hourly	60,356	−3	6	13	25
Daily	2526	−2	5	13	23
Barometric pressure (mmHg)					
Hourly	60,379	758	762	766	784
Daily	2525	758	762	766	781

Air pollution was measured hourly; total possible hours = 60,624; total possible days = 2,526.

a Concentrations missing from 21 January 1998 to 15 March 1999.

b Concentrations missing from 30 March 1997 to 15 October 1999.

**Table 2:** ORs for PAF associated with an interquartile range increase in the mean of pollutant lag hours.

Mean of pollutant	Interquartile range	Lags	No. of subjects	No. of PAF episodes	OR (95% CI)	*p*-Value
PM_2.5_ (μg/m^3^)	9.4	0	22	52	1.41 (0.82–2.42)	0.22
	7.8	0–23	22	47	1.13 (0.63–2.03)	0.68
Black carbon (μg/m^3^)	0.91	0	18	46	0.81 (0.42–1.56)	0.53
	0.83	0–23	18	46	1.46 (0.67–3.17)	0.34
NO_2_ (ppb)	13.2	0	28	90	1.21 (0.80–1.83)	0.37
	9.2	0–23	27	89	1.18 (0.79–1.76)	0.43
CO (ppm)	0.58	0	28	90	0.87 (0.56–1.37)	0.55
	0.51	0–23	28	90	0.71 (0.39–1.28)	0.25
SO_2_ (ppb)	4.9	0	28	90	1.02 (0.81–1.28)	0.87
	4.1	0–23	28	90	0.99 (0.71–1.39)	0.97
O_3_ (ppb)	21.7	0	28	90	2.08 (1.22–3.54)	0.007
	15.8	0–23	28	89	1.60 (0.89–2.89)	0.12
